# SARS-CoV-2-specific T cells in infection and vaccination

**DOI:** 10.1038/s41423-021-00743-3

**Published:** 2021-09-01

**Authors:** Antonio Bertoletti, Nina Le Bert, Martin Qui, Anthony T. Tan

**Affiliations:** 1 Programme in Emerging Infectious Diseases, Duke-NUS Medical School, Singapore, Singapore; 2 Singapore Immunology Network, A*STAR, Singapore, Singapore

**Keywords:** SARS-CoV-2, Vaccines, T cells, COVID-19, Immunological memory, Diagnostic markers

## Abstract

During viral infections, antibodies and T cells act together to prevent pathogen spread and remove virus-infected cells. Virus-specific adaptive immunity can, however, also trigger pathological processes characterized by localized or systemic inflammatory events. The protective and/or pathological role of virus-specific T cells in SARS-CoV-2 infection has been the focus of many studies in COVID-19 patients and in vaccinated individuals. Here, we review the works that have elucidated the function of SARS-CoV-2-specific T cells in patients and in vaccinated individuals. Understanding whether SARS-CoV-2-specific T cells are more linked to protection or pathogenesis is pivotal to define future therapeutic and prophylactic strategies to manage the current pandemic.

The characterization of the immunological events occurring during SARS-CoV-2 infection is proceeding at high speed. A multitude of studies have investigated different aspects of innate and adaptive immunity occurring both in animal models and in patients using classic or advanced technologies. The overall emerging picture of SARS-CoV-2 infection, as in many other viral infections, shows that SARS-CoV-2 can be efficiently controlled in most infected individuals through coordinated activation of the innate and adaptive components of the immune system. The impairment of IFN-α function mediated by increased production of anti-IFN-α autoantibodies has been associated with severe COVID-19 cases [1], while individuals able to control the infection without severe symptoms are able to rapidly mount a virus-specific antibody [2] and T cell response [3–5].

A more complex and less clear scenario is instead related to the pathogenesis of the disease, mainly characterized by severe lung immunopathology. The severity and length of local or systemic (i.e., cytokine storm) pathological events appears to be directly associated with viral load or with an immune response with uncoordinated features [6, 7]. In our recent review, we presented evidence that supports, in our opinion, the preferential association of SARS-CoV-2-specific T cells with protection [8]. Here, we will further expand this argument and discuss novel studies that have investigated the role of T cells in COVID-19 and vaccination. Understanding whether T cells are more linked to protection or pathogenesis is pivotal to define how current vaccines work and provide protection.

## SARS-CoV-2-specific T cells: protection or damage?

Although human studies do not offer direct proof of causality, several works have already shown that the rapid induction of SARS-CoV-2-specific T and B cell responses is associated with viral control and limited pathology (reviewed in [8–10]). Detailed analyzes of CD4 and CD8 T cells and B cells specific for different SARS-CoV-2 proteins showed that the coordinated presence of all the components of adaptive immunity was linked with limited disease severity [3]. Uncoordinated activation of humoral and cellular immunity was instead observed in elderly individuals, who more often develop severe pathological consequences [3].

### Protective role of SARS-CoV-2-specific T cells

**Fig. 1:**
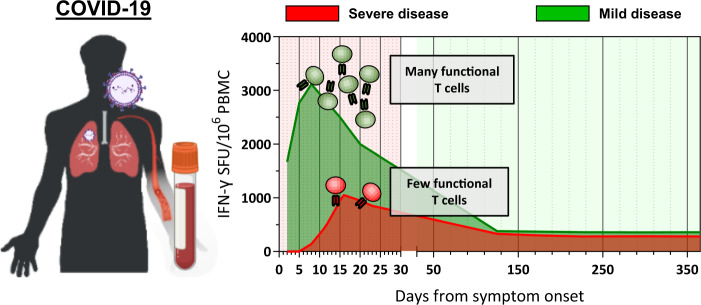
Kinetic of SARS-CoV-2 specific T cells. Schematic representation of the expansion and contraction kinetic of SARS-CoV-2 specific T cells in COVID-19 patients with mild or severe disease

The importance of the rapid deployment of the T cell response was also highlighted by our recent study, where we investigated the dynamic changes in virological and immunological parameters in a limited number of patients with symptomatic acute SARS-CoV-2 infection from disease onset to convalescence or death (12 patients in total, 3 with severe COVID-19) [4]. We quantified SARS-CoV-2 viral RNA in the respiratory tract in parallel with antibodies and circulating T cells specific for various structural (NP, M, ORF3a, and Spike) and nonstructural proteins (ORF7/8, NSP7 and NSP13). Early induction of IFN-γ-secreting SARS-CoV-2-specific T cells was present only in patients with mild disease and accelerated viral clearance [4] (Fig. 1). The rapid induction of immune responses was observed not only for T cells but also for antibodies. In a much larger study (229 patients analyzed), early induction of Spike-specific and neutralizing antibodies was associated with viral control and less severe disease [2].

It is important to point out that in pure quantitative terms, many studies have shown that the magnitude of antibodies and T cells was instead proportional to the severity of the disease [11, 12]. For example, a broader and quantitatively more robust SARS-CoV-2-specific T cell response has been demonstrated in patients with severe COVID-19 in comparison to those with mild COVID-19 [12–14]. However, the quantitative differences in SARS-CoV-2-specific T cell responses detectable after recovery were likely a mere consequence of their prolonged exposure to higher quantities of viral antigens typically attained in severe disease.

Supporting evidence that SARS-CoV-2-specific T cells mediate viral protection came from independent studies of cellular immunity in virally exposed but not infected individuals and in patients with asymptomatic SARS-CoV-2 infection. Virally exposed individuals, who are both PCR-negative and seronegative, possess SARS-CoV-2-specific T cells [15, 16]. A comparison of virus-specific T cells in symptomatic and asymptomatic patients revealed that the ability to mount a significant virus-specific T cell response is independent of the presence of symptoms. However, SARS-CoV-2-specific T cells in asymptomatic patients are functionally superior to those detected in patients with symptoms [17]. Two studies demonstrated this difference: one through the analysis of cytokine production (IFN-γ and IL-2) [17] and one other by single-cell analysis of the total population of T cells [18].

Recently, indirect evidence of the protective role of SARS-CoV-2-specific T cells has emerged from studies of patients with different immune defects or under specific immunosuppressive treatments. For example, patients with agammaglobulinemia developed only minor COVID-19 symptoms before full recovery [19–21]. In another study of COVID-19 patients with cancer, those with hematological cancers overall faced a relative survival disadvantage compared to those with solid cancers. Among these hematological patients with reduced humoral responses and deficient B cells, those with strong CD8 T cell responses demonstrated improved survival [22].

### Pathological role of SARS-CoV-2-specific T cells

If the presence and rapid deployment of highly functional SARS-CoV-2-specific CD8 and CD4 T cells is linked with control and mild/absent disease, several open questions concerning the role of virus-specific T cells during severe COVID-19 remain unsettled.

Studies that have analyzed SARS-CoV-2-specific T cell responses during severe COVID-19 cases are limited. However, a scenario of uncoordinated activation and possible exhaustion of SARS-CoV-2-specific T cells prevails [6, 7, 18, 23, 24]. In our recent longitudinal analysis of viral and immunological parameters in the early phase of COVID-19, we observed a markedly reduced and delayed rise in the frequency of SARS-CoV-2-specific T cells in patients who developed severe disease. Of note, in the patient who succumbed to the disease, we were unable to demonstrate the presence of circulating SARS-CoV-2-specific T cells despite the presence of high viral load and antibodies (mainly specific for nucleoprotein) [4]. The limitation of this study was, however, not only the low number of patients analyzed (only three patients with severe COVID-19) but also the fact that virus-specific T cells were analyzed in the circulation through IFN-γ ELISpot. This assay can detect only peripheral T cells secreting the Th1 cytokine IFN-γ. Hence, whether SARS-CoV-2-specific T cells in severe patients are localized in the lung or exhausted could not be answered.

Other studies showed increased frequencies of activated T cells and particularly CD8 effector T cells [13, 14, 18] in the peripheral blood of patients with severe COVID-19. However, the clearer event occurring in the blood of patients with severe COVID-19 is marked lymphopenia [25, 26], particularly of CD8 T cells [27], likely due to their robust recruitment into the lungs. Thus, the role of T cells in severe cases of COVID-19 should be studied primarily in the lung, the site of the major pathological events.

In this regard, studies have started to analyze in parallel the immunological profile present in the blood and lungs of patients with COVID-19 [28, 29]. For example, Szabo et al. obtained paired lung and blood samples from patients with severe disease [29]. Analysis was performed mainly by high-dimensional immune profiling using flow cytometry and scRNA-seq and, as such, did not investigate the quantity and function of SARS-CoV-2-specific T cells. Nevertheless, the data showed that younger patients with a benign disease have more lung resident T cells than older patients who ultimately succumb. Severe and persistent lung inflammation was instead associated with lower numbers of T cells and high quantities of myeloid cells (monocytes and macrophages). Myeloid cells are highly activated and secrete CCL-2, a chemokine that might promote ongoing inflammation. Importantly, the airway resident T cells (and not the peripheral T cells) detected in this study and associated with recovery were characterized by a phenotype of resident memory T cells. They were activated and characterized by the upregulated expression of perforin, granzyme and IFN-γ, a signature of classic functional antiviral-T cells [29]. A signature of T cell exhaustion was instead reported in another recent study that also analyzed airway resident immune cells. Here, the authors detected massive recruitment and activation of the myeloid cell compartment and T cells expressing “exhaustion signatures” proportional to disease severity [30].

Taken together, these data and other immunological studies directly investigating the events occurring in the lungs of patients with severe COVID-19 [28, 31] depict a scenario in which the immunopathogenesis of severe COVID-19 is characterized by inflammatory events (the activation and recruitment of myeloid cells into the airway, production of inflammatory cytokines and complement activation) not directly linked with SARS-CoV-2-specific T cell activation [32]. Such a scenario of parenchymal organ damage mediated preferentially by inflammatory myeloid cells has been demonstrated in other viral diseases, in which the virus does not have a direct cytopathic effect. For example, liver damage in chronic HBV infection is independent of the quantity of HBV-specific T cells. In contrast, HBV-specific T cells are more robust in patients who have limited or absent liver damage but a low level of viral replication [33, 34].

## Memory T cell response: protection after convalescence or vaccination

We have discussed the role of T cells in containing viral replication and in ensuring viral clearance without severe pathological sequelae. Next, we will explore the role of memory T cells induced by infection or by vaccination in providing protection from subsequent infection (sterilizing immunity) or disease control.

### SARS-CoV-2-specific T cells after natural infection

CD4 and CD8 T cells are present and can be directly detected in the blood of convalescent COVID-19 patients even up to 6 to 8–9 months post infection [35–39], irrespective of their disease severity [17, 38]. Quantitatively, the memory response is skewed toward more CD4/helper T cells than CD8/cytotoxic T cells despite their often equivalent frequencies immediately after infection [40]. As we have previously discussed, this is likely due to a more pronounced decline in circulating CD8 T cell frequency in the memory phase [8]. Whether such a circulatory decline in CD8 T cells might correspond to the establishment of a stable population of tissue resident T cells, which might be more important for immediate protection, is still unknown. These circulating memory CD8 T cells are composed of cells with a stem-cell-like memory phenotype, sustained polyfunctionality and proliferation capacity and are hence likely to mediate an anamnestic response [41]. Antibody levels wane more rapidly than T cells [42], but their decay is also not followed by the disappearance of SARS-CoV-2-specific B cells [43] or plasma cells [44]. Interestingly, Spike-specific B cells have been detected for longer periods of time even in elderly patients with rapidly declining levels of neutralizing antibodies [45].

The ability of SARS-CoV-2 infection to induce long-term adaptive immunity is expected. We have demonstrated that SARS-CoV-specific T cells were still detectable 17 years after infection [46]. In other viral infections, such as vaccinia, virus-specific memory B cells were detectable even 50 years after infection [47]. However, it is difficult to define the level of humoral and cellular immunity able to protect against infection or disease. Some insights can be derived from studies that analyzed the rate of re-infection and disease severity in COVID-19 convalescents. The results are, however, not univocal. A study involving healthcare workers in the UK showed that re-infection was extremely uncommon 6 months after previous infection [48]. Uncommon re-infection rates were also recently found in Italians 1 year after infection [49]. These studies support the idea that the natural induction of SARS-CoV-2 adaptive immunity confers a robust protective effect for at least 1 year, similar to what has also been reported in infections by other related coronaviruses [50]. On the other hand, the high rate of re-infection observed in Manaus (Brazil) [51] has challenged such a conclusion, but viral variants escaping the immunity elicited by the initial infection might explain this observation.

### SARS-CoV-2-specific T cells after vaccination

**Fig. 2:**
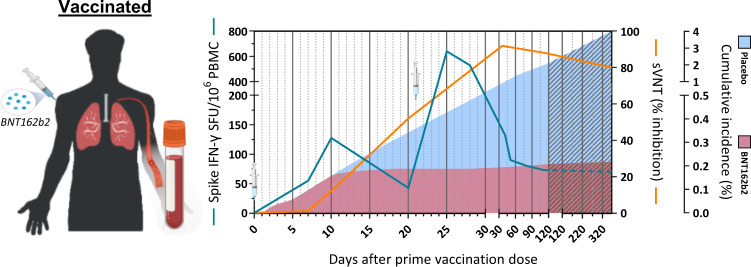
Schematic representation of Spike-specific T cell and neutralizing antibody response (surrogate virus neutralization assay) after BNT162b2 vaccination. Cumulative incidence of SARS-CoV-2 infection after vaccination with BNT162b2 (red shade) or placebo (blue shade) are also shown. Dotted lines and gray shaded area denotes the expected dynamics where data are not available at the moment of writing

The extraordinary pace of development and implementation of different vaccine preparations against SARS-CoV-2 have also provided the demonstration that priming of a virus-specific adaptive immune response protects against SARS-CoV-2 infection and COVID-19 severity. Observational studies of mRNA-, adenoviral-, protein-, and inactivated virus-based vaccines have shown that even with some differences in efficacy, all these different preparations have a significant effect in reducing disease severity and infection [52–59]. However, the relative contribution of humoral and cellular immunity in the protection process is still difficult to decipher. Prediction of antibody levels necessary to induce protection has been made [60, 61], but the contribution of T cells is still difficult to analyze for practical and perhaps conceptual reasons. Practically, the quantification of virus-specific T cells is technically more complex than serological analysis. Conceptually, T cells do not recognize the virus but only recognize virus-infected cells (CD8 T cells) or cells that have internalized viral antigens produced after infection (CD4 T cells). This means that T cells do not prevent infection, but their role is linked to a reduction in viral load within the host and consequently shorter infection with lower pathogenicity [62]. Nevertheless, some data are starting to show the importance of vaccine-induced Spike-specific T cells in protection. For example, a detailed analysis of humoral and cellular immunity after the initial BNT162b2 vaccine dose demonstrated that Spike-specific T cells and antigen-binding but not neutralizing antibodies were already detectable at day 10 in good quantities [63] (Fig. 2). Neutralizing antibodies started to appear in high titers only at day 21 and increased after the second dose. The interesting point of this longitudinal study is that the clinical trial of BNT162b2 vaccine efficacy showed that protection from symptomatic disease began approximately 10 days after the first dose [52]. These data therefore show that neutralizing antibodies are not the only correlate of protection. It is likely that Spike-specific T cells play, as in convalescent people, an important role in reducing viral replication and therefore limit the pathogenicity of infection.

The protective role of vaccine-induced T cells might contribute to vaccine efficacy against viral variants. Despite partial antibody escape, viral variants often cause only mild or asymptomatic disease in individuals who have completed a two-dose vaccination regimen [64, 65]. This could be explained by the virus’s inability to fully escape Spike-specific T cells. An analysis of the impact of the mutations on T cell recognition showed that in most vaccinated individuals [66], the variants of concern are recognized by Spike-specific T cells induced by mRNA [66–69] and adenoviral vaccines [70] designed with the ancestral viral strain. Vaccine-induced Spike-specific T cells are clearly multispecific and capable of recognizing different regions of the Spike protein [68, 69]. Although some mutations might alter single T cell specificities, they do not escape the whole repertoire of Spike-specific T cells.

### Heterogeneity of vaccine-induced SARS-CoV-2-specific T cells

The characteristics of vaccine-induced Spike-specific T cells warrant further discussion. Advanced studies capable of providing a detailed profile of Spike-specific T cells have been performed mainly in individuals vaccinated with mRNA- or adenovirus-based vaccines. Therefore, we have limited the discussion to data obtained with these vaccine preparations.

mRNA vaccines induce Spike-specific T cells that preferentially produce IL-2 and IFN-γ [71] and recognize different regions of Spike. CD4 and CD8 T cells seem to be present in roughly equivalent numbers [72], although initial data suggested that CD4 T cells were predominant [67, 68, 71]. We recently observed that at 3 months after vaccination, the mean quantity of Spike-specific T cells was equivalent to what has been detected in convalescent patients at a similar time after SARS-CoV-2 infection [73].

Of note, Spike-specific T cells are highly heterogeneous in different vaccine recipients not only in terms of specificity but also in quantitative terms. Indeed, since T cells recognize short sequences of viral antigens (epitopes) derived from the processing of viral antigens associated with HLA-class I or HLA-class II molecules, it is unsurprising that each individual will be characterized by a distinct fingerprint of T cells that varies in relation to its HLA-class I and Class II profile [74]. Recent analysis of the number of T cell epitopes already mapped in SARS-CoV-2 infection revealed the extent of such multispecificity [75].

The quantity of Spike-specific T cells has also been observed to vary by at least 1 logarithmic value (i.e., 200–300 spots or 2000–3000 spots per million CD4 or CD8 T cells) irrespective of the methods used for their detection. This variability was found not only in vaccinated naïve individuals [69, 72, 73] but also in those who were already primed by SARS-CoV-2 infection [66, 71, 76, 77], in recipients above 80 years of age [78] and in those under immunosuppressive treatments [79]. On average, vaccination in COVID-19 convalescent individuals is more effective than in naïve individuals, and a robust Spike-specific T cell response is induced after a single dose [66, 71, 76, 77]. Senior individuals older than 80 years mount, on average, weaker IFN-gamma and IL-2 CD4 T cell responses than younger individuals [78]. Moreover, vaccinations in patients with B cell deficiency due to anti-CD20 therapy modified their vaccine-induced Spike-specific T cells. A CD4 follicular helper T cell deficit and preferential CD8 T cell induction have been reported [79]. However, despite these differences detectable in these specific groups, considerable variations in the quantity of Spike-specific T cells are induced even in a cohort of individuals of similar age and the absence of other pathologies [69, 72, 73].

The quantitative heterogeneity of cellular immunity has also been overlooked after natural infection. Differences in both humoral and cellular responses in females versus males or in subjects of different ages or with concomitant pathologies are present and have been attentively studied [3, 80]. However, we are progressively observing marked quantitative differences in adaptive immunity levels both in convalescent and vaccinated individuals, irrespective of their age, sex or severity of SARS-CoV-2 infection [73, 81]. These marked differences were detected in terms of antibody levels and T cell responses after natural infection [17, 81, 82]. Furthermore, the distinct components of adaptive immunity appear to be independently regulated, since, for example, the level of antibodies cannot predict the quantity of Spike-specific T cells, particularly in the late convalescent phase (8–9 months) [35, 81, 82].

This difference in the magnitude of T cell responses (both in convalescent and vaccinated) and the lack of correlation between antiviral antibody production and T cell frequency could cause difficulties in the analysis of correlates of protection. In addition, current mRNA- and adenovirus-based vaccines elicit immune responses confined to the Spike protein. However, the potentially protective role of T cells specific for other structural and nonstructural proteins in protection will also have to be evaluated. The idea that polyantigenic vaccines might actually exert greater control over viral variants than monoantigenic (i.e., Spike-based) has its rationale [64, 83]. From this perspective, we think that the role of T cells specific for nonstructural proteins involved in the replication machinery of the virus (coded by the ORF1ab gene) will have to be carefully evaluated. T cells specific for proteins coded by ORF1ab have been found in high numbers both in recovered COVID-19 patients [84] and in individuals who might have been only exposed to SARS-CoV-2 [85] or related coronaviruses [46].

## Concluding remarks

In this review, we discussed the numerous data that support the important role of CD4 and CD8 virus-specific T cells during SARS-CoV-2 infection. We have mainly considered quantitative aspects, and we acknowledge that immunological analysis in blood samples might have limitations for understanding the role of T cells in protection and pathology. Studies of SARS-CoV-2-specific T cells in mucosal tissue will need to be expanded. On the other hand, the modification of the epidemiology of the infection caused by mass vaccination campaigns and the overall astonishing success of different vaccines calls for a better evaluation of the correlates of protection that need to be defined to manage the COVID-19 pandemic. The heterogeneity in the level of T cell immunity induced in both convalescent and vaccinated individuals attests to the intricacy of such an analysis, requiring the implementation of large prospective studies where both antibody and T cell levels are measured. The technical complexity of SARS-CoV-2-specific T cell measurements has, however, often limited this analysis, and its implementation to methods that can be routinely performed in large numbers, such as ELISpot or cytokine release assays [73, 86, 87], might start to provide such data [88]. We see a future where T cell studies might guide, through the collection of precise functional and quantitative data, the management of future vaccination campaigns that will hopefully transform SARS-CoV-2 infection into a mild nuisance for the entire world.

## Data Availability

Data analyzed in this study were a re-analysis of existing data, which are openly available at locations cited in the reference section.
